# Molecular Characterization and Clonal Analysis of Carbapenem‐Resistant *Acinetobacter baumannii*: Insights Into Biofilm‐Related Gene Coexistence in Clinical Isolates

**DOI:** 10.1155/bmri/2304337

**Published:** 2026-01-30

**Authors:** Mahtab Hadadi, Bahram Nasr Esfahani, Arezoo Mirzaei, Sharareh Moghim

**Affiliations:** ^1^ Bacteriology and Virology Department, School of Medicine, Isfahan University of Medical Sciences, Isfahan, Iran, mui.ac.ir

**Keywords:** *Acinetobacter baumannii*, antimicrobial resistance, biofilm-related genes, carbapenemase, rep-PCR

## Abstract

The emergence of multidrug‐resistant *Acinetobacter baumannii* (MDR *A. baumannii*) and biofilm‐producing ability have become a worldwide serious concern. This study is aimed at investigating the clonal relationships, coexistence of carbapenemase‐resistant and biofilm‐related genes, and biofilm biomass capacity in 57 *A. baumannii* isolates obtained from patients in intensive care units (ICUs). Antibiotic resistance patterns to 11 antibiotics were determined using the disc diffusion test. The minimum inhibitory concentrations (MICs) of imipenem and colistin were evaluated by the microdilution method. All isolates were subjected to PCR for the detection of carbapenemase‐ and biofilm‐related genes and examined for the biofilm‐forming ability using crystal violet staining methods. The clonality relationship was identified by rep‐PCR. Overall, 49 (86%) isolates were characterized as extensively drug‐resistant (XDR) with a high MIC for imipenem. Eight isolates were resistant to colistin (MIC>64 *μ*g/mL). Additionally, 86.21% of isolates were strong biofilm formers, which correlated with the PDR phenotype. All isolates carried at least three genes related to biofilm formation. Genotypically, 100% of isolates had *bla*_OXA−51-like_, *bla*_OXA−24−like_, and *bla*_TEM_ genes, followed by *bla*_VIM_ (61.4%), *bla*_OXA-23-like_ (24.6%), *bla*_SHV_ (1.8%), and *bla*_KPC_ (1.8%), whereas *bla*_CTX-M_ and *bla*_OXA-58-like_ genes were not found in the isolates. The rep‐PCR analysis identified 10 distinct genotypes, among which GTG Type 3 showed a significant correlation with strong biofilm formation. Moreover, the greatest number of colistin‐resistant isolates (MIC>64 *μ*g/mL) were located in this cluster. This study highlights the emergence of PDR *A. baumannii* strains carrying a variety of *β*‐lactamase and biofilm‐related genes in ICUs, underscoring the urgent need for improved infection control measures and antimicrobial stewardship programs to address the spread of these formidable pathogens.

## 1. Introduction

*Acinetobacter baumannii* (*A. baumannii*) is among the leading causes of hospital‐acquired infections, mainly in patients admitted to the intensive care unit (ICU) and those with immunocompromised conditions [1]. Carbapenems are considered the antibiotics of choice for treating infections caused by multidrug‐resistant (MDR) *A. baumannii* strains [2–4]. In the last 3 decades, the widespread use of broad‐spectrum antibiotics has led to the rapid development of MDR and extensively drug‐resistant (XDR) *A. baumannii* strains [1, 5, 6]. Carbapenem resistance in *A. baumannii* is mainly related to chromosomally produced carbapenemase, mostly encoded by *bla*_OXA-51-like_, or acquired oxacillinases, chiefly encoded by *bla*_OXA-23-like_ [7]. However, other resistance mechanisms, including enzymatic degradation of antibiotics, reduced expression of certain outer membrane channel‐forming proteins (porins), and the activity of efflux pumps, result in *β*‐lactam resistance in *A. baumannii* [8]. In Iran, the high prevalence (80%) of carbapenem‐resistant *A. baumannii* (CRAB) has become a serious problem in clinical settings [9].

Biofilm formation is one of the crucial virulence factors in *A. baumannii* that greatly supports the survival of *A. baumannii* in healthcare settings by increasing the resistance of the bacteria to antibiotics [10]. Several virulence factors, including outer membrane protein A (OmpA), biofilm‐associated protein (Bap), and pilus‐mediated colonization (Csu), are key elements in the biofilm formation of *A. baumannii*. OmpA is involved in the adhesion of *A. baumannii* [11]. Bap, as a surface protein, is a critical factor in initiating infection and biovolume [12]. A prevalent pilus, Csu, is vital in cell adhesion and biofilm formation [13]. Moreover, the presence of the global regulator of *A. baumannii* pathogenicity, *BfmRS*, and the *pga*A gene encoding poly‐*β*‐1,6‐N‐acetylglucosamine (PNAG), which is the major compound of the extracellular matrix in the biofilm of *A. baumannii*, as well as their association with CRAB require further investigation [13]. The association of these virulence factors with CRAB remains unclear, as previous studies reported inconsistent results: Qi L. et al. reported that *A. baumannii* can exhibit high levels of antibiotic resistance despite forming weak biofilms [14], whereas Bardbani et al. and Sheriff et al. have shown a positive correlation between antimicrobial resistance and strong biofilm‐forming ability in MDR *A. baumannii* isolates [10, 15].

This study is aimed at investigating the molecular resistance determinants, biofilm genes coexistence, and genotypic relationships among *A. baumannii* isolates in Isfahan teaching hospitals. We analyzed the potential correlations between biofilm formation capacity, biofilm‐associated virulence factors, and antimicrobial resistance genes. We also assessed the epidemiological association of isolates using repetitive element PCR (rep‐PCR), one of the most efficient methods for strain discrimination and prediction of circulating isolates through cluster analysis of banding patterns [16, 17].

## 2. Materials and Methods

### 2.1. Sample Collection and Bacterial Identification

In the present study, 57 clinical *A. baumannii* isolates were collected from February 2023 to November 2023 from four main university hospitals in Isfahan, Iran. The isolates were obtained from patients admitted to ICUs. All isolates were cultured on MacConkey agar (Merck, Germany) for 18 h at 37°C. Nonlactose‐fermenting colonies were identified as *A. baumannii* by standard conventional methods, including oxidase, citrate, Oxidation–Fermentation (OF), and motility tests, followed by PCR of the unique species‐specific *bla*_OXA-51_ gene using the primer described in Table 1 [23]. The study was approved by the medical ethics committee of Isfahan University of Medical Sciences under the Approval Number (IR.MUI.MED.REC.1402.092).

**Table 1:** Primer sequences.

**Target gene**	**Primer sequence (5** ^′^ **–3** ^′^ **)**	**Amplicon size (bp)**	**References**
*bla*_OXA-51-like_	F: TAATGCTTTGATCGGCCT TG R: TGGATTGCACTTCATCTTGG	353	[18]
*bla*_OXA−24−like_	F: GGTTAGTTGGCCCCCTTAAA R: AGTTGAGCGAAAAGGGGAT	246	[18]
*bla*_OXA-23-like_	F: GATCGGATTGGAGAACCAGA R: ATTTCTGACCGCATTTCCAT	501	[18]
*bla*_OXA-58-like_	F: AAGTATTGGGGCTTGTGCTG R: CCCCTCTGCGCTCTACATAC	599	[18]
*bla*_TEM_	F: AGTATTCAACATTTCCGTGTC R: GCTTAATCAGTGAGGCACCTATC	850	[19]
*bla*_VIM_	F: GATGGTGTTTGGTCGCATA R: CGAATGCGCAGCACCAG	390	[20]
*bla*_SHV_	F: ATGCGTTATATTCGCCTGTG R: GTTAGCGTTGCCAGTGCTCG	862	[19]
*bla*_KPC_	F: CGTCTAGTTCTGCTGTCTTG R: CTTGTCATCCTTGTTAGGCG	798	[20]
*bla*_CTX-M_	F: TTTGCGATGTGCAGTACCAGTAA R: CGATATCGTTGGTGGTGCCATA	544	[19]
*bap*	F: TGCTGACAGTGACGTAGAACCACA R: TGCAACTAGTGGAATAGCAGCCCA	184	[12]
*ompA*	F: GTTAAAGGCGACGTAGACG R: CCAGTGTTATCTGTGTGACC	578	[21]
*csuE*	F: TTGGCTTTAGCAAACATGACCT R: TTGCGGGGAAAGTCCATTATTT	754	[22]
*bfmR*	F: GGATCTTGTGGTCTTGGATGTC R: GATAAAATACGGCCAGCGTTTG	557	[22]
*bfmS*	F: CACGTATTCGCTTTGGTACAGA R: GGCTATCATCTAAACGGGCAAA	990	[22]
*pgaA*	F: GCAAATGAATCCTTCCGATCCT R: GTTTTGAGTCGTTTTTCGCCAT	460	[22]

### 2.2. Antibiotic Susceptibility Testing

The susceptibility pattern of the isolates against different classes of antibiotics including piperacillin (10 *μ*g), imipenem (10 *μ*g), ceftazidime (30 *μ*g), amikacin (30 *μ*g), doxycycline (30 *μ*g), gentamicin (30 *μ*g), amoxicillin–sulbactam (10 *μ*g), cefepime (10 *μ*g), trimethoprim/sulfamethoxazole (1.25/23.75 *μ*g), ciprofloxacin (10 *μ*g), and tetracycline (30 *μ*g) (BBL, United States) was performed using the disk diffusion method according to the Clinical Laboratory Standards Institute (CLSI 2024) guidelines [24]. *A. baumannii* ATCC 19606 was used as a positive control. The isolates were categorized as MDR when they were found to be resistant to at least three classes of antibiotics. XDR isolates were resistant to all antibiotic groups except polymyxin (colistin and polymyxin B), whereas pan‐drug resistant (PDR) *A. baumannii* isolates were resistant to nearly all antibiotic groups, including colistin [25]. The minimum inhibitory concentration (MIC) for imipenem and colistin was determined by the microbroth dilution method based on CLSI 2024 guidelines [24].

### 2.3. Microtiter Plate Biofilm Formation Assay

The crystal violet staining method was used to assess the biofilm formation capacity of clinical isolates as described previously [22]. An overnight culture of *A. baumannii* isolates was prepared in trypticase soy broth (TSB, Merck, Germany) and adjusted to a 0.5 McFarland unit (approximately 1.5 × 10^8^ CFU/mL). Two hundred microliters of each diluted bacterial suspension (1:20) in TSB were dispensed in triplicate into 96‐well flat‐bottom microtiter plates and incubated at 37°C for 24 h. Then, planktonic cells were removed by gentle aspiration, and the biofilms were washed with phosphate‐buffered saline (PBS, pH 7.4). The biomass was stained with 0.1% (*w*/*v*) crystal violet for 15 min at room temperature. Excess stain was removed by washing, and the bound dye was solubilized with 33% (*v*/*v*) glacial acetic acid for 15 min at 37°C. Biofilm formation was quantified by measuring the optical density at 630 nm (OD_630_) using a microplate reader. *A. baumannii* ATCC 19606 and uninoculated TSB medium were used as a reference strain and negative control, respectively. The clinical isolates were classified as nonbiofilm formers when *OD* ≤ *OD*_c_, weak biofilm formers when *O* *D*_c_ < *O* *D* ≤ (2 × *O* *D*_c_), moderate biofilm formers when 2 × *O* *D*_c_ < *O* *D* ≤ (4 × *O* *D*_c_), and strong biofilm formers when *O* *D* > (4 × *O* *D*_c_) [12].

### 2.4. Detection of *β*‐Lactamase and Biofilm‐Related Genes

Genomic DNA was extracted from bacterial isolates by the standard boiling method as previously described [26] and assessed by NanoDrop spectrophotometry (A260/A280 ratio 1.8–2.0, DNA sample concentration of approximately 50–100 ng/*μ*L). This ensured adequate DNA quality and reproducibility of the rep‐PCR patterns. PCR amplification was performed using Taq DNA polymerase (Amplicon, Denmark) with specific primer sets (Table 1) to detect *β*‐lactamase resistance genes, including *bla*_OXA-23-like_, *bla*_OXA-24-like_, *bla*_OXA-58-like_, *bla*_TEM_, *bla*_VIM_, *bla*_SHV_, *bla*_KPC_, and *bla*_CTX-M_, and biofilm‐associated virulence factors including *omp*A, *bap*, *csu*E, *bfm*S, *bfm*R, and *pga*A. The amplification was carried out under the following conditions: initial denaturation at 94°C for 5 min; followed by 30 cycles of denaturation (94°C for 45 s), annealing (56°C–62°C for 45 s, temperature optimized for each primer pair), with an extension time of 1 min at 72°C to ensure complete amplification of fragments up to 1.5 kb; and a final extension at 72°C for 5 min. DNA from a sequenced *Escherichia coli* isolate was used as a carbapenemase‐positive control [19].

### 2.5. Rep‐PCR for Molecular Typing and Clonal Analysis of CRAB

To determine the clonality of isolates, rep‐PCR was performed using a REP (5 ^′^‐GTGG TGGTGGTGGTG‐3 ^′^) primer [19]. The PCR was carried out under the following conditions: initial denaturation for 3 min at 95°C; followed by 30 cycles of 45 s at 94°C, primer annealing for 30 s at 50°C, and extension for 1 min at 72°C; and final extension for 5 min at 72°C. PCR products were visualized by electrophoresis on a 1.5% agarose gel [19]. The 1.5% agarose gels were run for 2 h at 60 V. A 100‐bp plus DNA ladder (ThermoFisher Scientific, Germany) was used as a size marker. The band patterns were analyzed using GelJ software (v. 2.0). A stringent 100% similarity cutoff was applied for rep‐PCR profile analysis. Isolates demonstrating complete genetic profile identity (100% band pattern similarity) were classified as clonally related and grouped within the same rep‐PCR cluster. Discriminatory index (D) was calculated using Simpson′s diversity index to evaluate the typing method′s resolution at varying similarity thresholds [19].

### 2.6. Statistical Analysis

Nei′s distances were used to determine genotypic diversity among the isolates, as previously outlined by Weir [27]. The discriminatory power of the rep‐fingerprinting method was assessed by calculating Simpson′s diversity index, following previously established methodologies [28] The Mann–Whitney *U* test or the Kruskal–Wallis *H* test was used when comparing the biofilm‐forming capacity between two groups or among three groups, respectively. Statistical analyses were conducted using GraphPad Prism Version 9. Statistical significance was set at a *p* value of ≤ *0.05*.

## 3. Results

### 3.1. Sample Collection and Bacterial Identification

A total of 57 *A. baumannii* clinical isolates were collected from the ICUs of four tertiary care teaching hospitals, with a mean age of 45 years (*n* = 34 male and *n* = 23 female). All isolates were confirmed as *A. baumannii* by standard biochemical identification methods and PCR.

### 3.2. Antibiotic Susceptibility Testing

The evaluated antimicrobial resistance pattern showed a 100% resistance to piperacillin, cotrimoxazole, ciprofloxacin, imipenem, ceftazidime, and ampicillin–sulbactam, followed by amikacin (98.2%), cefepime (96.5%), doxycycline (96.5%), tetracycline (86%), and gentamicin (77.2%) (Figure 1a). The MICs of isolates against two antibiotics, imipenem and colistin, were determined. All isolates were found to be resistant to imipenem (MIC >256 *μ*g/mL). Notably, 14% (8/57) of the isolates were resistant to colistin, with a MIC greater than 64 *μ*g/mL. Of the 57 clinical isolates, 86% (49/57) were classified as XDR, and 14% (8/57) were grouped as PDR (Table 2).

Figure 1Frequency of antibiotic resistance and carbapenemase gene in *Acinetobacter baumannii* clinical isolates. (a) Antimicrobial resistance patterns of isolates against different classes of antibiotics. The sensitivity of *A. baumannii* clinical isolates to various antibiotics was determined using the Kirby–Bauer method in accordance with CLSI guidelines. The stacked bar chart illustrates the resistance percentage of 57 isolates to each tested antibiotic. (b) Distribution of carbapenem resistance (CR) genes among clinical isolates. The bar graph illustrates the percentage of clinical isolates harboring different CR genes.

**Figure:**
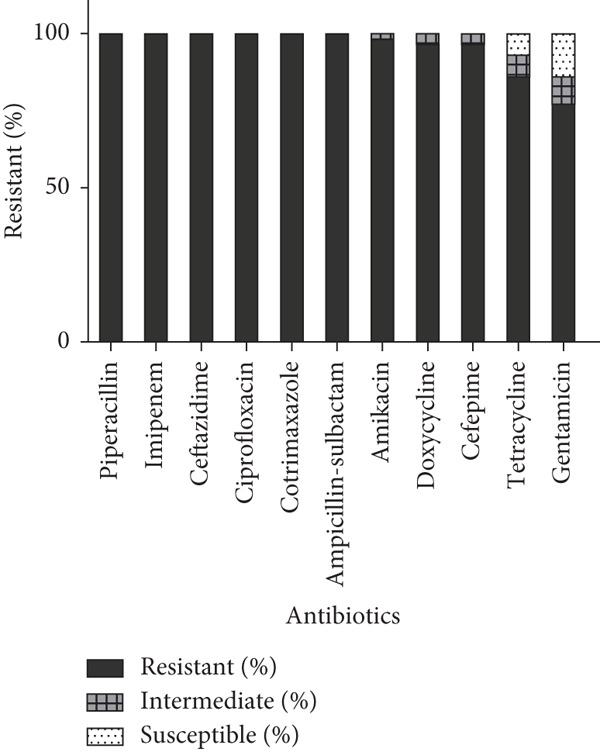
(a)

**Figure:**
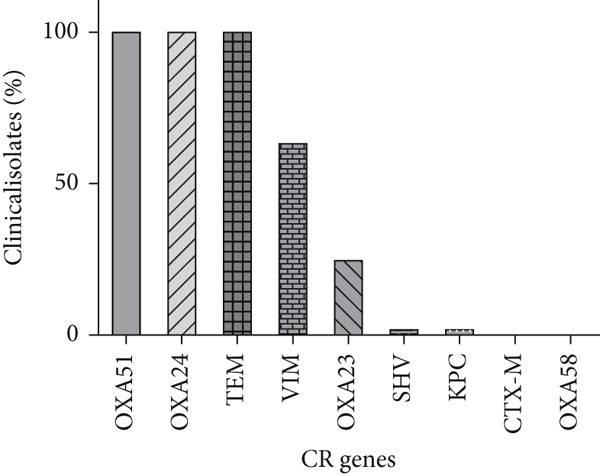
(b)

**Table 2:** Molecular characterization of resistance determinants, MICs, and clonal distribution of *Acinetobacter baumannii* isolates from ICU.

**Isolates**	**CR genes**	**Biofilm genes**	**Biofilm capacity**	**XDR/PDR**	**MIC of IMP**	**MIC of COL**	**Rep-PCR profile**
AB.48	*OXA51, OXA24, TEM*	*ompA, bap, pgaA*	M	XDR	> 256	2	GTG01
AB.38	*OXA51, OXA24, TEM, VIM*	*ompA, bap, csuE, pgaA, bfmR, bfmS*	S	XDR	> 256	2	GTG01
AB.36	*OXA51, OXA24, TEM, VIM*	*ompA, bap, csuE, pgaA, bfmR, bfmS*	S	PDR	> 256	> 64	GTG01
AB.35	*OXA51, OXA24, TEM, VIM*	*ompA, bap, csuE, pgaA, bfmR, bfmS*	S	PDR	> 256	> 64	GTG01
AB.28	*OXA51, OXA24, TEM, VIM*	*ompA, bap, pgaA*	S	XDR	> 256	2	GTG01
AB.22	*OXA51, OXA24, TEM, VIM*	*ompA, bap, csuE, pgaA, bfmR, bfmS*	S	XDR	> 256	2	GTG01
AB.17	*OXA51, OXA24, TEM, VIM*	*ompA, bap*	S	XDR	> 256	2	GTG01
AB.16	*OXA51, OXA24, TEM, VIM*	*ompA, bap*	S	XDR	> 256	2	GTG01
AB.14	*OXA51, OXA24, TEM, VIM*	*ompA, bap, csuE, pgaA, bfmR, bfmS*	S	XDR	> 256	2	GTG02
AB.5	*OXA51, OXA24, TEM*	*ompA, bap, csuE, bfmR*	S	XDR	> 256	2	GTG02
AB.50	*OXA51, OXA24, TEM*	*ompA, bap, pgaA, bfmR*	S	XDR	> 256	1	GTG03
AB.37	*OXA51, OXA24, TEM*	*ompA, bap, csuE, pgaA, bfmR, bfmS*	S	PDR	> 256	> 64	GTG03
AB.34	*OXA51, OXA24, TEM, VIM*	*ompA, bap, csuE, pgaA, bfmR, bfmS*	S	PDR	> 256	> 64	GTG03
AB.32	*OXA51, OXA24, TEM*	*ompA, bap, pgaA, csuE, bfmS*	S	PDR	> 256	> 64	GTG03
AB.30	*OXA51, OXA24, TEM, VIM*	*ompA, bap, pgaA*	S	XDR	> 256	1	GTG03
AB.25	*OXA51, OXA24, TEM, VIM*	*ompA, bap, pgaA*	S	XDR	> 256	1	GTG03
AB.19	*OXA51, OXA24, TEM, VIM*	*ompA, bap, csuE, bfmS*	S	XDR	> 256	1	GTG03
AB.18	*OXA51, OXA24, TEM, VIM*	*ompA, bap, csuE, pgaA, bfmR, bfmS*	S	XDR	> 256	1	GTG03
AB.15	*OXA51, OXA24, TEM, VIM*	*ompA, bap*	S	XDR	> 256	1	GTG03
AB.11	*OXA51, OXA24, TEM, OXA23*	*ompA, bap, bfmS*	S	XDR	> 256	1	GTG04
AB.9	*OXA51, OXA24, TEM*	*ompA, bap, csuE, pgaA, bfmR, bfmS*	S	XDR	> 256	2	GTG04
AB.7	*OXA51, OXA24, TEM*	*ompA, bap*	S	XDR	> 256	1	GTG04
AB.4	*OXA51, OXA24, TEM, VIM, OXA23*	*ompA, bap*	S	XDR	> 256	1	GTG04
AB.33	*OXA51, OXA24, TEM, VIM*	*ompA, bap, csuE, pgaA, bfmR, bfmS*	S	PDR	> 256	> 64	GTG05
AB.31	*OXA51, OXA24, TEM, VIM*	*ompA, bap, csuE, pgaA, bfmR*	S	XDR	> 256	2	GTG05
AB.29	*OXA51, OXA24, TEM*	*ompA, bap, pgaA, bfmR*	S	XDR	> 256	1	GTG05
AB.12	*OXA51, OXA24, TEM, VIM*	*ompA, bap, bfmS*	M	XDR	> 256	1	GTG05
AB.10	*OXA51, OXA24, TEM, VIM*	*ompA, bap, bfmR*	S	XDR	> 256	2	GTG05
AB.24	*OXA51, OXA24, TEM, VIM*	*ompA, bap, bfmS*	S	XDR	> 256	1	GTG06
AB.21	*OXA51, OXA24, TEM, VIM*	*ompA, bap, csuE, pgaA, bfmR, bfmS*	S	XDR	> 256	1	GTG06
AB.8	*OXA51, OXA24, TEM, VIM, OXA23*	*ompA, bap*	S	XDR	> 256	2	GTG06
AB.6	*OXA51, OXA24, TEM*	*ompA, bap, csuE, bfmR, bfmS*	S	XDR	> 256	2	GTG06
AB.2	*OXA51, OXA24, TEM, VIM*	*ompA, bap*	S	XDR	> 256	2	GTG06
AB.1	*OXA51, OXA24, TEM, VIM*	*ompA, bap, bfmR*	S	XDR	> 256	2	GTG06
AB.45	*OXA51, OXA24, TEM*	*ompA, bap, pgaA, bfmR, bfmS*	S	XDR	> 256	2	GTG07
AB.44	*OXA51, OXA24, TEM*	*ompA, bap, pgaA*	S	XDR	> 256	1	GTG07
AB.27	*OXA51, OXA24, TEM, VIM*	*ompA, bap, pgaA, bfmR*	W	XDR	> 256	2	GTG07
AB.20	*OXA51, OXA24, TEM, VIM*	*ompA, bap*	S	XDR	> 256	1	GTG07
AB.13	*OXA51, OXA24, TEM, OXA23*	*ompA, bap*	M	PDR	> 256	> 64	GTG07
AB.3	*OXA51, OXA24, TEM, VIM, OXA23*	*ompA, bap*	S	PDR	> 256	> 64	GTG07
AB.56	*OXA51, OXA24, TEM, VIM, OXA23*	*ompA, bap, pgaA*	M	XDR	> 256	2	GTG08
AB.47	*OXA51, OXA24, TEM, OXA23*	*ompA, bap, csuE, pgaA, bfmR, bfmS*	S	XDR	> 256	2	GTG08
AB.46	*OXA51, OXA24, TEM, VIM*	*ompA, bap, csuE, pgaA, bfmR*	S	XDR	> 256	2	GTG08
AB.43	*OXA51, OXA24, TEM, VIM*	*ompA, bap, pgaA, bfmS*	S	XDR	> 256	2	GTG08
AB.40	*OXA51, OXA24, TEM*	*ompA, bap, pgaA, bfmR*	S	XDR	> 256	2	GTG08
AB.26	*OXA51, OXA24, TEM*	*ompA, bap*	S	XDR	> 256	2	GTG08
AB.57	*OXA51, OXA24, TEM, KPC*	*ompA, bap, pgaA, bfmR*	M	XDR	> 256	2	GTG09
AB.52	*OXA51, OXA24, TEM, VIM*	*ompA, bap, pgaA, bfmR*	S	XDR	> 256	1	GTG09
AB.49	*OXA51, OXA24, TEM, OXA23*	*ompA, bap, pgaA, bfmR*	S	XDR	> 256	1	GTG09
AB.42	*OXA51, OXA24, TEM, VIM, OXA23*	*ompA, bap, bfmR*	M	XDR	> 256	1	GTG09
AB.41	*OXA51, OXA24, TEM, VIM, OXA23*	*ompA, bap, csuE, pgaA, bfmR, bfmS*	S	XDR	> 256	2	GTG09
AB.39	*OXA51, OXA24, TEM, VIM*	*ompA, bap, pgaA, bfmR*	S	XDR	> 256	2	GTG09
AB.23	*OXA51, OXA24, TEM, VIM, OXA23*	*ompA, bap*	S	XDR	> 256	1	GTG09
AB.55	*OXA51, OXA24, TEM, OXA23*	*ompA, bap, pgaA, bfmR*	M	XDR	> 256	2	GTG10
AB.54	*OXA51, OXA24, TEM, VIM, OXA23*	*ompA, bap, csuE, pgaA, bfmR, bfmS*	S	XDR	> 256	1	GTG10
AB.53	*OXA51, OXA24, TEM, OXA23*	*ompA, bap, csuE, pgaA, bfmR, bfmS*	S	XDR	> 256	1	GTG10
AB.51	*OXA51, OXA24, TEM, SHV*	*ompA, bap, pgaA, bfmR*	S	XDR	> 256	1	GTG10

Abbreviations: COL, colistin; CR genes, carbapenems resistance genes; IMP, imipenem; M, moderate; MIC, minimum inhibitory concentration; PDR, pan‐drug resistant; S, strong; W, weak; XDR, extensively drug resistant.

### 3.3. Molecular Detection of *β*‐Lactamase Genes

**Figure 2:**
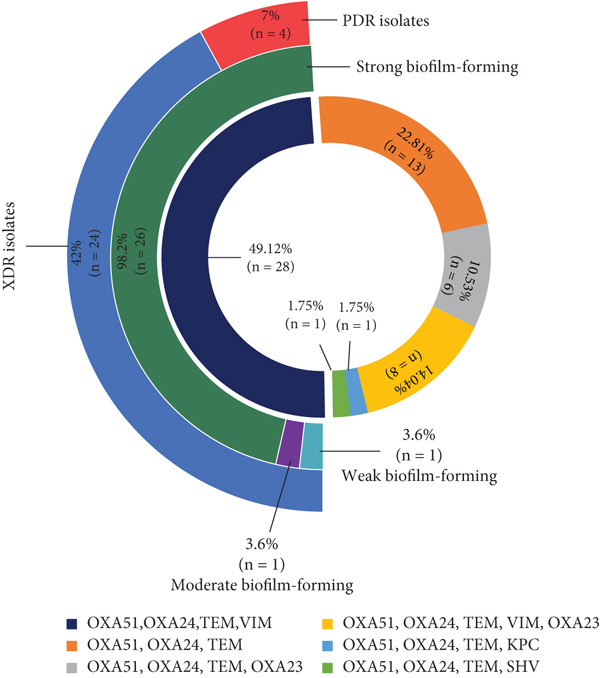
Frequency of a set of carbapenem‐resistant (CR) genes with biofilm formation capacity among *Acinetobacter baumannii* isolates showing differences in resistance and biofilm‐forming strength. Approximately 85.7% of the isolates harboring OXA‐51, OXA‐24, and TEM genes exhibited extensive drug resistance (XDR) with strong biofilm capacity. XDR: extensively drug‐resistant; PDR: pan‐drug‐resistant.

Molecular identification of carbapenemase genes revealed that all isolates harbored *bla*_TEM_ and *bla*_OXA−24−like_. The prevalence of other carbapenemase genes was as follows: *bla*_VIM_ 63.2% (36/57), *bla*_OXA−23−like_ 24.6% (14/57), *bla*_SHV_ 1.8% (1/57), and *bla*_KPC_ 1.8% (1/57) (Figure 1b). In this study, *bla*_CTX-M_ and *bla*_OXA-58-like_ were not detected in any of the tested isolates. Nearly half of the isolates (49.12%) carried a combination of four resistance genes (*bla*_OXA-51-like_, *bla_OXA-24-like_*, *bla_TEM_*, and *bla_VIM_*). Notably, almost all isolates carrying this gene profile (98.3%) demonstrated strong biofilm‐forming ability, and this combination was predominantly associated with PDR isolates (Figure 2). A significant association was observed between the coexistence of the antimicrobial resistance genes *bla_OXA-24-like_*, *bla_TEM_*, and *bla_VIM_*, and the strong biofilm‐forming capacity of the bacterial isolates (*p* = 0.014). The following most frequent gene pattern (22.81%) comprised *bla*_OXA-51-like_, *bla_OXA-24-like_*, and *bla_TEM_*. Although isolates harboring this gene set also tended to form strong biofilms, the correlation was less prominent compared with the four‐gene combination. Other, less common resistance gene patterns, such as those including *bla*_OXA-23-like_, *bla*_KPC_, or *bla*_SHV,_ showed greater variability in biofilm‐forming potential, with some isolates displaying moderate or weak biofilm production. The frequency of potent biofilm‐forming isolates among the other gene sets did not show any significant correlation.

### 3.4. Biofilm Formation Capacity in *A. baumannii* Isolates

Biofilm‐forming assay results showed that 86.21% (49/57) of *A. baumannii* clinical isolates produced strong biofilms, 12.07% (7/57) exhibited moderate biofilms, and one isolate (1.72%) was detected as weak biofilm‐forming. To determine whether colistin‐resistant isolates could produce more biofilms, the biomass of colistin‐resistant and colistin‐intermediate isolates was compared. The Mann–Whitney comparison of optical density values (OD_630_) of biofilm formation between colistin‐resistant and intermediate groups (*p* = 0.01) confirmed a statistically significant difference (Figure 3a).

Figure 3Role of biofilm‐associated genes and antimicrobial resistance in biofilm formation capacity of clinical *Acinetobacter baumannii*. (a) Comparison of biofilm formation capabilities between colistin‐resistant (MIC≥4) and colistin‐intermediate (MIC<2) clinical isolates of *A. baumannii*. Data were presented as median with interquartile range from three biological replicates. The Mann–Whitney *U* test was performed to compare biofilm production (OD_630_) between colistin‐intermediate and colistin‐resistant isolates, *p* = 0.01. (b) Distribution of biofilm‐associated genes among *A. baumannii* isolates with different biofilm‐forming capacities. The bar plot illustrates the number of isolates carrying each biofilm‐related gene according to their biofilm production strength, as determined by the crystal violet microtiter plate assay. The Kruskal–Wallis test between biofilm‐forming groups (strong, moderate, and weak) in each biofilm‐related gene confirmed a statistically significant difference.

**Figure:**
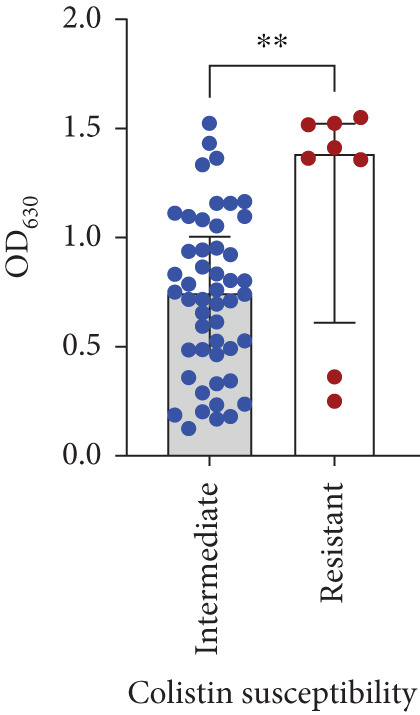
(a)

**Figure:**
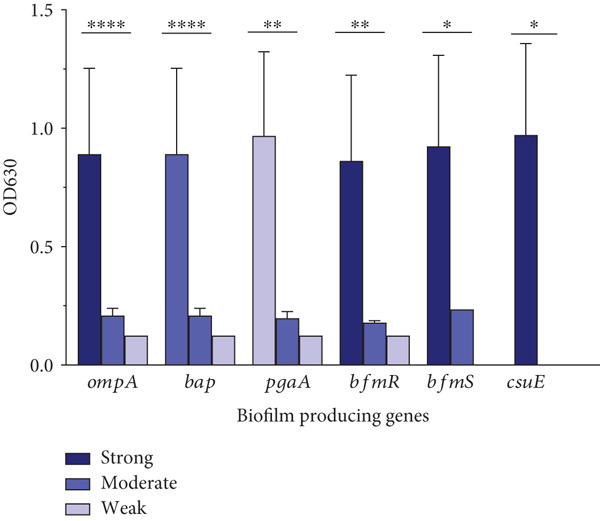
(b)

### 3.5. Biofilm‐Associated Genes

The PCR of biofilm‐associated genes revealed that the *omp*A and *bap* were evident in all examined isolates, followed by the frequency of *pga*A 63.2% (36/57), *bfm*R 56.1% (32/57), *bfm*S 40.4% (23/57), and *csu*E 36.8% (21/57). A significant relationship was observed between the distribution of biofilm‐related genes and biofilm biomass production (Figure 3b).

### 3.6. Rep‐PCR for Molecular Typing and Clonal Analysis of CRAB

**Figure 4:**
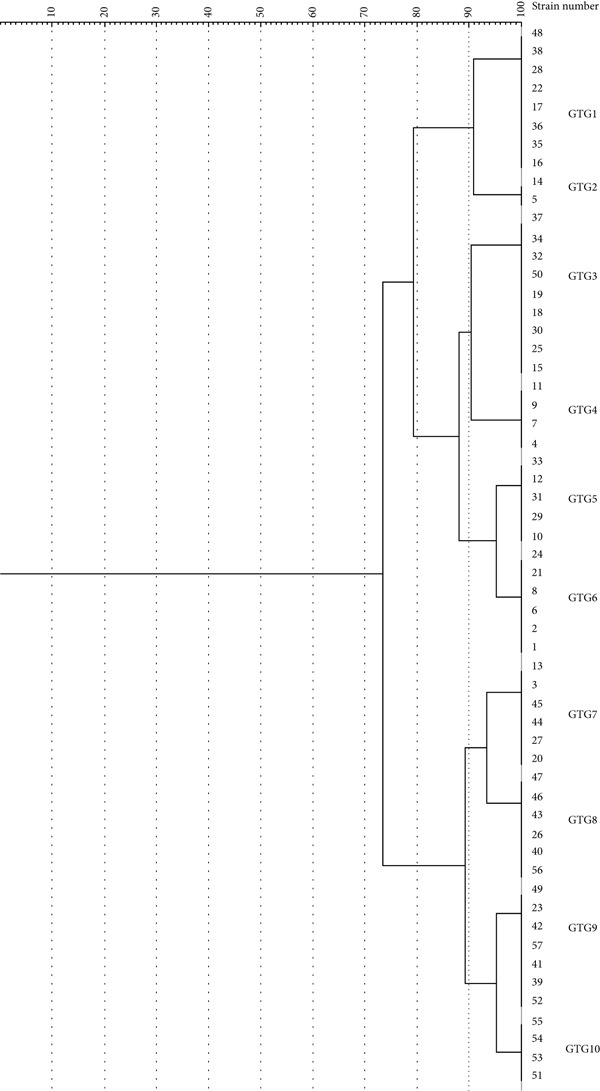
Dendrogram of similarity among *Acinetobacter baumannii* clinical isolates from patients in intensive care units (ICUs) by rep‐PCR. Clustering was performed using the unweighted pair group method with arithmetic mean (UPGMA) in GelJ software (Version 3.0). The isolates were grouped into 10 distinct genotypes (GTG1–GTG10), indicating high genetic diversity among carbapenem‐resistant strains.

Rep‐PCR fingerprints from 57 isolates generated amplification profiles with 2–10 bands, ranging from 250 to 1.5 kb. This amplification identified 10 distinct (GTG) types (GTG1‐GTG10) among the isolates (Figure 4). Cluster analysis of the rep‐PCR banding patterns demonstrated significant discriminatory power, as evidenced by a Simpson′s diversity index (D) of 0.904 (95% CI: 0.86–0.94).

## 4. Discussion

The elevated rates of resistant *A. baumannii* represent a significant threat in ICU settings, and polymyxins such as colistin have proven to be the last effective antibiotic of choice for treating *A. baumannii-*related infections [29]. This issue becomes increasingly complicated due to the bacteria′s ability to survive in challenging environments through the formation of biofilms [12]. In Iran, epidemiological studies revealed a concerning escalation in XDR *A. baumannii* prevalence, increasing from 62.8% in 2014 to 97.3% in 2018 [30]. Our study demonstrated that 100% (57) of clinical isolates met the criteria for XDR. Notably, imipenem MIC values demonstrated a broad resistance spectrum (100%) among isolates, representing a concerning escalation from previous national reports. A systematic review of antimicrobial resistance patterns in the Middle East in 2016 revealed substantial regional variation in imipenem‐resistant *A. baumannii* (IRAB) prevalence [31]. Although Iran reported 55% IRAB incidence, neighboring countries demonstrated significantly higher rates: Pakistan (100%), Turkey (98%), United Arab Emirates (76%), and Saudi Arabia (63%) [31]. This regional pattern was further confirmed by a 2020 meta‐analysis of 122 clinical studies, which reported an overall imipenem resistance rate of 74.2% among *A. baumannii* isolates [32]. We found that 14.03% (8/57) of clinical isolates were colistin resistant, consistent with emerging resistance patterns observed throughout the region [33].

The molecular analysis revealed the universal presence of *bla*_OXA-24-like_ and *bla*_TEM_ genes among all isolates, with variable co‐occurrence patterns of additional resistance determinants. Almost half of the strains (49.12%) carried four carbapenemase genes (*bla*_OXA-51-like_, *bla*_OXA-24-like_, *bla*_TEM_, and *bla*_VIM_), whereas 14% harbored five carbapenemase genes, including *bla*_OXA-23-like_, along with the previous set. The high frequency of multicarbapenemase genotypes in ICU‐acquired XDR strains presents substantial clinical challenges and enhances the potential for horizontal dissemination of resistance genes [34]. Moreover, quantitative biofilm assays demonstrated a 100% prevalence of biofilm‐forming capacity among isolates, with 86.21% classified as strong producers, aligning with those currently investigated in other reports from Iran [35] and Egypt [10]. The convergence of extensive carbapenemase production and robust biofilm formation capacity in these strains is particularly concerning, as both traits synergistically contribute to treatment failure and environmental persistence [12].

This study investigated the potential relationship between genotypic resistance profiles and the biofilm‐forming ability. The results showed that the presence of additional carbapenemase genes (*bla*_VIM_, *bla*_OXA-23-like_, *bla*_SHV_, or *bla*_KPC_) was strongly associated with enhanced biofilm formation, suggesting a direct relationship between increased resistance gene burden and robust biofilm production. Furthermore, this finding highlights a potential synergistic relationship between a certain set of antibiotic resistance genes and biofilm‐associated phenotypes. The coexistence of these carbapenemase genes likely contributes to enhanced adaptive capacity, favoring bacterial survival under antibiotic pressure and within biofilm communities. Our results support previous studies linking biofilm formation to antibiotic resistance, emphasizing the combined role of genetic resistance factors and phenotypic adaptations in the persistence and treatment failure of *A. baumannii* [21, 36, 37]. Contrary to our findings, several reports indicate that the emergence of strong biofilm producers is not dependent on the existence of antimicrobial resistance genes [14, 38]. These discrepancies underscore the complexity and controversy of this relationship, highlighting the need for larger sample sizes and further investigation into the genetic linkage between antibiotic resistance and biofilm genes, beyond those examined, to fully understand the underlying mechanism. The comparison of optical density (OD_630_) values among isolates carrying different biofilm‐associated genes revealed that the presence of these genes was significantly correlated with the strength of biofilm. Isolates harboring *omp*A and *bap* genes exhibited markedly higher OD values compared with those with weaker biofilm phenotypes, suggesting their strong contribution to surface adherence and biofilm maturation. Similarly, *pga*A, *bfm*R, *bfm*S, and *csu*E were also associated with increased biofilm production, although at varying levels of significance. Previous studies have supported a strong positive correlation between the presence of *omp*A, *bap*, and *csu*E genes and biofilm formation [10, 39, 40]. Furthermore, the coexistence of more than five biofilm‐related genes (*omp*A, *bap*, *csu*E, *pga*A, *bfm*R, and *bfm*S) showed a strong correlation with heightened biofilm formation, indicating a significant link between a higher number of biofilm‐related genes and stronger biofilm‐producing ability. Since *A. baumannii* is a nosocomial pathogen, this relationship may be a key factor in the persistence, transmissibility, and treatment resistance of nosocomial infections. Our results also revealed a notable association between biofilm production and resistance to colistin in the isolates. This observation aligns with earlier studies reporting a strong correlation between biofilm production and colistin resistance in *A. baumannii* clinical isolates [40, 41]. Nevertheless, some studies have reported that certain colistin‐resistant *A. baumannii* strains exhibited reduced biofilm formation compared with their colistin‐susceptible counterparts [42]. The strong association between the *bla_OXA-51-like_*, *bla_OXA-24-like_*, *bla_TEM_*, and *bla_VIM_* gene combination and both the PDR phenotype and biofilm formation suggests that these genes may play a role in promoting persistence and virulence. It is plausible that the selective pressure imposed by antibiotic exposure in clinical environments has favored isolates that are both resistant to a broad range of antibiotics and capable of producing robust biofilms. Our findings offer new regional insights into gene coexistence patterns and correlations with biofilm strength.

In this study, rep‐PCR was used to discriminate the clonal relationship among the isolates. The widespread presence of *bla*_OXA-24-like_ and *bla*_TEM_ across all 10 detected types suggested the circulation of carbapenemase resistance genes among the isolates. All identified types demonstrated a consistent genotypic profile, with biofilm‐related genes, such as *omp*A and *bap*, present in all *A. baumannii* isolates from ICUs in various reference hospitals in Isfahan. This study highlights that GTG1, GTG2, GTG3, and GTG10 show significant diversity in both resistance and biofilm‐related genes. Nevertheless, colistin‐resistant isolates were found to be disseminated in four genetically distinct clusters, which is a concerning issue due to the possibility of horizontal gene transfer (HGT) as a driving mechanism [43]. The collection of these clones from various reference hospitals in our region raises serious concerns about the potential widespread dissemination of resistant pathogens. This situation poses a significant threat to public health systems and efforts to control infections in hospitals. We selected rep‐PCR as a rapid and cost‐effective alternative suitable for the aims of the present investigation, particularly in resource‐limited settings. Future studies incorporating multilocus sequence typing (MLST) or whole‐genome sequencing (WGS) analyses are warranted to elucidate better the population structure, genetic diversity, and evolutionary relationships of *A. baumannii* isolates at both local and international levels.

## 5. Conclusion

In conclusion, these findings suggest that certain combinations of carbapenemase genes may serve as molecular markers of high‐risk clones characterized by both extensive drug resistance and strong biofilm‐forming ability, posing a considerable challenge for infection control and therapeutic management in healthcare settings. The dual threat of extensive drug resistance and universal biofilm formation necessitates the development of novel therapeutic strategies combining anti‐biofilm agents with next‐generation antimicrobials.

## Conflicts of Interest

The authors declare no conflicts of interest.

## Funding

This study was supported by Isfahan University of Medical Sciences (10.13039/501100003970, No. 340247).

## Data Availability

The data that support the findings of this study are available from the corresponding author upon reasonable request.
